# Transcriptome Analysis Reveals Molecular Underpinnings of Common Carp (*Cyprinus carpio*) Under Hypoxia Stress

**DOI:** 10.3389/fgene.2022.907944

**Published:** 2022-05-20

**Authors:** Ning Suo, Zhi-Xiong Zhou, Jian Xu, Ding-Chen Cao, Bi-Yin Wu, Han-Yuan Zhang, Peng Xu, Zi-Xia Zhao

**Affiliations:** ^1^ State Key Laboratory of Marine Environmental Science, College of Ocean and Earth Sciences, Xiamen University, Xiamen, China; ^2^ Key Laboratory of Aquatic Genomics, Ministry of Agriculture and Rural Affairs, Chinese Academy of Fishery Sciences, Beijing, China; ^3^ Heilongjiang River Fisheries Research Institute, Chinese Academy of Fishery Sciences, Harbin, China

**Keywords:** common carp, hypoxia stress, transcriptome, immune response, HIFs

## Abstract

As an essential environmental factor that affects the economic benefits of aquaculture, hypoxia is one of the urgent problems to be solved in the aquaculture fish breeding industry. Common carp (*Cyprinus carpio*) is a critical economic fish in China, and at present, there are many breeding strains of common carp with different character advantages in China, including Hebao red carp (*C. carpio* var *wuyuanesis*) and Songpu mirror carp (*C. carpio* var *specularis*). Even if the environmental adaptation of common carp is generally strong, the genetic background of hypoxia tolerance in different strains of common carp is unclear yet. This study tested the hypoxia tolerance of Songpu minor carp, Hebao red carp, and their hybrid F1 population by an acute hypoxia treatment. Muscle and liver tissues were used for transcriptome sequencing analysis to identify the key factors for hypoxia tolerance and explore the potential genetic mechanism for breeding high hypoxia tolerance in common carp. The comparative transcriptomic analysis revealed abundant hypoxia response-related genes and their differential regulation mechanism in these two tissues of different common carp strains under acute hypoxia, including immune response, cellular stress response, HIFs (hypoxia-inducible factors), MAP kinase, iron ion binding, and heme binding. Our findings will facilitate future investigation on the hypoxia response mechanism and provide a solid theoretical basis for breeding projects in common carp.

## Introduction

Dissolved oxygen is a major limiting factor affecting fish survival, and its concentration in water concentration is affected by the photosynthesis of aquatic plants and the respiration of aquatic organisms (Abdel-Tawwab et al., 2019). Low dissolved oxygen in the water will cause fish hypoxia response, which has maximum negative effects on fish growth, immune response, and cellular stress response (Jiang et al., 2017). In Atlantic salmon (*Salmo salar*), chronic hypoxia modulates the expression of immune genes to make differences in immune response (Kvamme et al., 2013). In chronic hypoxia-stressed adult rainbow trout (*Oncorhynchus mykiss*), basal catecholamine secreted increasingly, and chromaffin cells enhanced responsiveness to adapt to constant stress (Montpetit and Perry, 1998). With global warming and water pollution becoming more serious, hypoxia has gradually caused more economic severe losses in the aquaculture industry, so it is necessary to understand the mechanism of hypoxia adaptation for fish breeding.

Transcriptional regulation plays a vital role in hypoxia-stressed fish. Numerous immune-relevant genes were differently expressed in hypoxia-stressed large yellow croaker (*Larimichthys crocea*) spleen and head kidney (Mu et al., 2020a). Besides, growing evidence shows that hypoxia regulates many innate immunological processes, such as phagocytosis of pathogens, antigen presentation, production of chemokines, and antimicrobial factors (Harris et al., 2014). Moreover, in rainbow trout, hypoxia stress caused cellular stress response, especially the up-regulated expression of *hsp70* (Fuzzen et al., 2011). Hsp70 is a classic stress protein, and its expression is significantly up-regulated in response to stresses such as heat, hyperosmolarity (Oehler et al., 1998), ischemia (Sanders et al., 1995), and acute hypoxia. Moreover, the hypoxia-inducible transcription factors are also critical for hypoxia-stressed organisms (Semenza, 2010). In tilapia (*Oreochromis niloticus*) heart, many hypoxia-responsive genes were identified to be involved in the hypoxia-stressed, including *eno* and *egln* (Xia et al., 2018). Besides, a previous study indicated that HIF (hypoxia-inducible factor) proteins control hypoxia response and regulate development, organ differentiation, and circadian rhythm in zebrafish (*Danio rerio*) (Pelster and Egg, 2018). Iron is also critical to hypoxia-stressed fish, participating in cellular respiration and oxygen transfer (Bury and Grosell, 2003). Moreover, iron’s transport and steady state are critical for HIF proteins, especial for HIF-2α protein (Shah and Xie, 2014).

The common carp (*Cyprinus carpio*) is the earliest monoculture fish globally, having a long domestication history and cultural value (Mason, 1984). At present, common carp is one of the most widely farmed fish globally, accounting for 10% of annual global freshwater aquaculture production (FAO, 2017). On the other hand, due to long-term geographical isolation, adaptation, and human domestication, China has a wealth of carp germplasm resources with diverse genetic characteristics (Hu et al., 2017). Hebao red carp (*C. carpio* var *wuyuanesis*) and Songpu mirror carp (*C. carpio* var *specularis*) are the primary common carp breeding strains distributed in China, and there are significant differences in body shape, growth, body color, scale pattern, and hypoxia tolerance among these strains of carp. Dhillon et al. found that there was significant variation in hypoxia tolerance among 10 closely related groups of cyprinids, among which crucian carp (*Carassius carassius*) and goldfish (*Carassius auratus*) were the most hypoxia-tolerant species, and common carp, koi (*C. carpio*), and bighead carp (*Aristichthys nobilis*) were the second hypoxia-tolerant group (Dhillon et al., 2013). According to our study, there was also significant variation in our two strains of common carp and their hyprid F1 carp hypoxia tolerance. Songpu mirror carp, as a selective strain of German mirror carp (*C. carpio mirror*), has stronger stress resistance than Hebao red carp (Li et al., 2018) and is more tolerant of hypoxia. Also, as a tetraploid species, common carp has clear heterosis and the traits in which hybrids express heterosis, including survival, growth, and tolerance to specific diseases (Wohlfarth, 1993). Our study, the hybrid F1 population, showed moderate hypoxia tolerance ability, indicating heterosis in hypoxia tolerance. Besides testing hypoxia tolerance among two strains of common carp and their hybrid F1 carp by an acute hypoxia stress experiment, we also used muscle and liver for transcriptome sequencing analysis to identify the key genes and regulatory pathways for hypoxia adaptation and explored the potential molecular mechanism, which provided a solid theoretical basis for the ongoing breeding projects in common carp.

## Materials and Methods

### Common Carp Strains Used in Hypoxia Treatment

Hebao red carp, Songpu mirror carp, and hybrid F1 carp used in this study came from three full-sib families, and all of them were bred in Hulan hatchery station of Heilongjiang River Fisheries Research Institute, Chinese Academy of Fishery Sciences by artificial insemination in May 2018. The hybrid F1 population was bred using Songpu mirror carp as the female parent and Hebao red carp as the male parent, half-sib with the other two strains. All three strains were raised in a 1t pond until 3 months old, and healthy individuals with a body weight of 197 ± 16 g were selected for hypoxia treatment.

### Hypoxia Treatment and Sampling

Before the hypoxia treatment, fish were raised at 25°C for 7 days in a 1-m^3^ indoor poor and fed twice a day. During this time, the dissolved oxygen concentration was controlled at 6.43 ± 0.39 mg/L; 7 days later, 10 fish were randomly selected from each of the two common carp strains and their hybrid F1 carp and were evenly divided into normoxia and hypoxia treatment groups. Feeding was stopped for both groups during the whole treatment. The hypoxia group stopped oxygenation after the experiment began and measured dissolved oxygen every 15 min using an Orion Star A223 RDO meter (ThermoFisher, United States); the decreased rate of the dissolved oxygen in the water was recorded (Supplementary Table S1). The normoxia group maintained 6.0 ± 0.1 mg/L dissolved oxygen by an aerator. The hypoxia group went through a 24-h hypoxia treatment whose dissolved oxygen maintained at 1.02 ± 0.06 mg/L.

After anesthetizing with 200 mg/L MS222, a total of 60 individuals were sampled, including i) 10 Hebao red carp from hypoxia treatment, ii) 10 Hebao red carp from normoxia control, iii) 10 Songpu mirror carp from hypoxia treatment, iv) 10 Songpu mirror carp from normoxia control, v) 10 hybrid F1 carp from hypoxia treatment, and vi) 10 hybrid F1 carp from normoxia control. Approximately 2 g muscle and 2 g liver tissues were collected from each individual and flash-frozen in liquid nitrogen, separately. We collected 2-g dorsal muscles and livers of each individual from two groups, then quickly frozen the tissue in liquid nitrogen, and stored it at -80°C at last for subsequent RNA extraction.

### RNA Extraction

Sixty samples, including 30 oxygen control samples and 30 hypoxia-stressed samples from two tissues of two common carp strains and their hybrid F1 carp, were collected for RNA extraction. RNA was extracted using the phenol/chloroform method (Chomczynski and Sacchi, 1987). Shredded tissue was added to DEPC water to 100ul and mixed with phenol/chloroform (1:1) under normal temperature. After centrifuging for 15 min, an equal volume of chloroform was added to the supernatant. Then, we centrifuged the supernatant again and added 2.5 times the volume of anhydrous ethanol. After 24 h precipitation at −80°C, RNA was collected by brief centrifugation, washed twice with 70% ethanol, air-dried, and resuspended in milli-Q water. Next, RNA concentrations were quantified using spectroscopy by Nanodrop 2000 (Thermo Scientific) and checked by 1.5% agarose gel electrophoresis stained with ethidium bromide for integrity. Finally, 60 RNA samples met the quality requirement for library construction; the qualified RNA samples were sent to Annoroad (Beijing, China) to construct RNA-seq libraries and sequencing with PE-150 paired-end strategy on the Hiseq platform.

### Transcriptome Analysis

After deleting low-quality bases (quality <20), sequencing adapters, short reads, and unpaired reads, 148,476,367,800 clean reads were used to analyze by the following steps. Firstly, clean reads were mapped to a common carp reference genome (PRJNA510861) by hisat2-2.1.0 (Kim et al., 2015). Then, stringtie-1.3.4 (Pertea et al., 2015) was used to assemble, merge, and quantify the transcripts. To identify differentially expressed genes (DEGs), the R package DESeq2 (Love et al., 2014) was used with the threshold of “*p* values <0.05 and |log2 Fold Change| ≥ 2". In order to get an overview of different functional classes in two strains of common carp and their hybrid F1 carp, the R package clusterProfiler (Yu et al., 2012) was used to perform Gene Ontology (GO) analysis. Moreover, the metabolic pathways were constructed based on the Kyoto Encyclopedia of Genes and Genomes (KEGG) database also using the clusterProfiler package.

### qRT-PCR Analysis

In this study, 18 DEGs were randomly selected for qRT-PCR to verify the accuracy of RNA-seq. The cDNA was obtained according to the method described previously (Bai et al., 2020) using the RevertAid First Strand cDNA Synthesis Kit, and primers used for qPCR are shown in Supplementary Table S2. The thermal cycling program was set as 95°C for 1 min, followed by 40 cycles of 95°C for 15 s and 60°C for 115 s. The β-actin gene was used as the internal control. The relative expression levels of target genes were analyzed using the comparative threshold (CT) cycle method (Livak and Schmittgen, 2001).

## Results

### Transcriptome Sequencing

**FIGURE 1:**
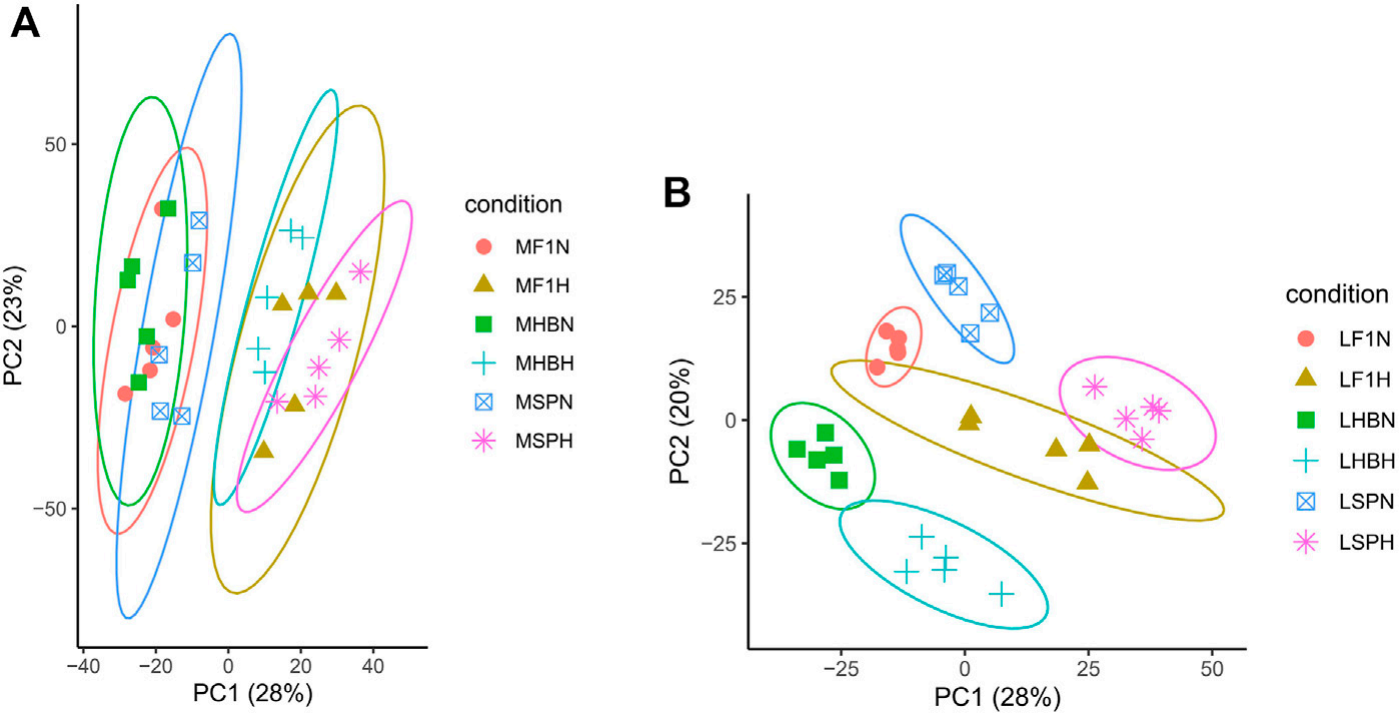
Principal component analysis of gene expression in the muscle **(A)** and liver **(B)** samples (M, Muscle; L, Liver; HB, Hebao red carp; SP, Songpu mirror carp; F1, Hybrid F1 carp; H, Hypoxia-stressed samples; N, Normal oxygen samples; the samples used were MHBN1, MHBN2, MHBN3, MHBN4, MHBN5, MHBH1, MHBH2, MHBH3, MHBH4, MHBH5; MSPN1, MSPN2, MSPN3, MSPN4, MSPN5, MSPH1, MSPH2, MSPH4, MSPH5, MSPH6; MF1N1, MF1N2, MF1N3, MF1N4, MF1N5, MF1H1, MF1H2, MF1H3, MF1H4, MF1H5; LHBN1, LHBN2, LHBN3, LHBN5, LHBN6, LHBH1, LHBH2, LHBH3, LHBH4, LHBH; LSPN1, LSPN3, LSPN4, LSPN5, LSPN6, LSPH1, LSPH2, LSPH3, LSPH5, LSPH6; LF1N1, LF1N2, LF1N3, LF1N4, LF1N5, LF1H1, LF1H3, LF1H4, LF1H5, LF1H6). Different colors correspond to carp strains and different shades to different treatments.

To obtain an overview of the two strains of common carp and their hybrid F1 carp gene expression profile, 153,679,206,500 raw reads of 60 samples were obtained. After filtering low-quality bases (quality <30), sequencing adapters, short reads, and unpaired reads, 148,476,367,800 clean reads were retained, and the mapping ratio of a reference genome for each library ranged from 90.49 to 93.33% (Supplementary Table S3). The results of PCA indicated that 51% of the variations in muscle gene expression and 48% of the variations in liver gene expression were captured by PC1 and PC2, respectively (Figure 1). The inter-group and intra-group variations in gene expression were captured by PC1 and PC2, respectively. There were obvious distinctions between the normal oxygen and hypoxia-stressed muscle groups; there was less distance between different common carp strain samples and more distance between the normal oxygen and hypoxia-stressed muscle samples, indicating that the variants in muscle were primarily caused by hypoxia stress instead of strain. On the contrary, liver samples clustered separately due to hypoxia stress and strain, indicating that there were more variants in the liver than in muscle, and the liver plays more critical roles in response to hypoxia stress. No matter in the hypoxia-stressed muscle or liver samples, the hybrid F1 populations were clustered in the middle of Hebao red carp and Songpu mirror carp.

### DEGs of Two Common Carp Strains and Their Hybrid F1 Carp Muscle Under Hypoxia

**FIGURE 2:**
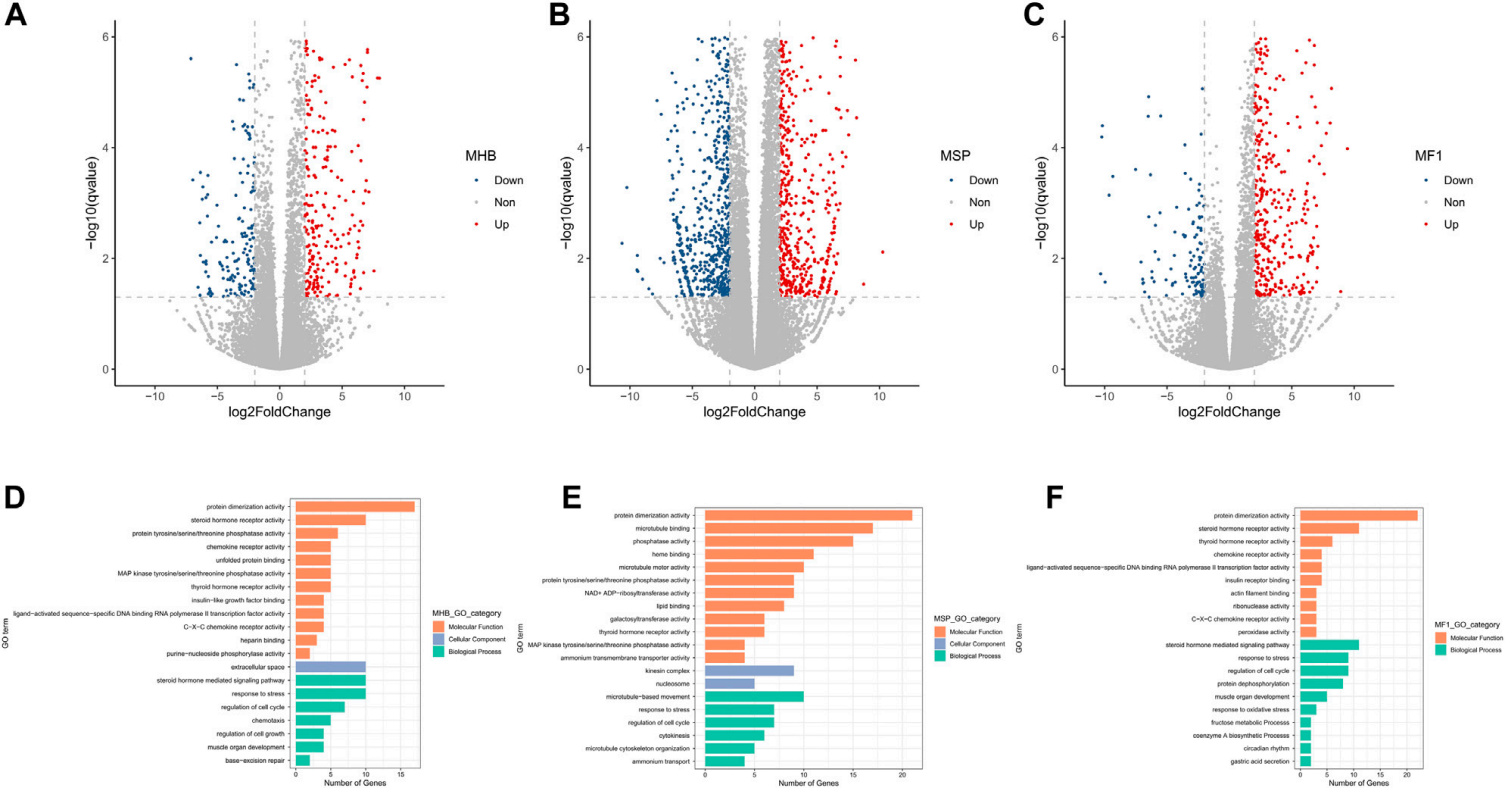
Volcano plot of log2 fold change and -log10 (FDR) of the differentially regulated genes demonstrated that most of the genes were up-regulated in all three strains of carp muscle. **(A)** Volcano plot for Hebao red carp muscle DEGs (MHBN1, MHBN2, MHBN3, MHBN4, MHBN5, MHBH1, MHBH2, MHBH3, MHBH4, MHBH5). **(B)** Volcano plot for Songpu mirror carp muscle DEGs (MSPN1, MSPN2, MSPN3, MSPN4, MSPN5, MSPH1, MSPH2, MSPH4, MSPH5, MSPH6). **(C)** Volcano plot for hybrid F1 carp muscle DEGs (MF1N1, MF1N2, MF1N3, MF1N4, MF1N5, MF1H1, MF1H2, MF1H3, MF1H4, MF1H5). GO enrichment analysis for genes differentially expressed between normoxia and hypoxia groups. **(D)**. Significantly enriched GO terms are shown for hypoxia-stressed Hebao red carp muscle. **(E)**. Significantly enriched GO terms are shown for hypoxia-stressed Songpu mirror carp muscle. **(F)**. Significantly enriched GO terms are shown for hypoxia-stressed hybrid F1 carp muscle.

The differentially expressed genes (DEGs) between the normoxia and hypoxia groups were identified by the threshold of “> 2-fold change in expression level (FDR <0.05),” leading to 478 up-regulated and 179 down-regulated genes in Hebao red carp muscle, 881 up-regulated and 697 down-regulated genes in Songpu mirror carp muscle, and 617 up-regulated and 125 down-regulated genes in hybrid F1 carp muscle, respectively (Figures 2A,B,C; Supplementary Tables S4–6).

Thirty-one GO terms were significantly enriched for hypoxia-stressed Hebao red carp muscle (Supplementary Table S7). Among the top 20 significantly enriched GO terms for Hebao red carp muscle, the most significant term was “response to stress” in the biological process (BP) category related to cellular stress response (Figure 2D). Besides, there were another two GO terms associated with cellular stress response, including “regulation of cell cycle” and “base-excision repair,” both in the BP category. Four of the top 20 enriched terms were related to immune response, such as “C–X–C chemokine receptor activity” and “chemokine receptor activity” in the molecular function (MF) category. There were 45 significant GO terms enriched in Songpu mirror carp muscle (Supplementary Table S8), and the most significant term was “regulation of cell cycle” in the BP category. “Heme binding” in the MF category directly involved in oxygen transport was also enriched (Figure 2E). For their hybrid F1 population, 35 GO terms were significantly enriched (Supplementary Table S9), and the most significant GO term was also the “regulation of cell cycle” in the BP category (Figure 2F). Among two strains of common carp and their hybrid F1 carp, “regulation of cell cycle,” “response to stress,” “protein dimerization activity,” and “thyroid hormone receptor activity” were all enriched, which suggested the crucial roles that the common carp muscle played in response to hypoxia stress.

TNF signaling pathway, Kaposi sarcoma-associated herpesvirus infection, IL-17 signaling pathway, C-type lectin receptor signaling pathway, osteoclast differentiation, and circadian rhythm were enriched in two strains of common carp and their hybrid F1 carp, which suggested that immune response plays essential roles in the common carp muscle responding to hypoxia stress (Supplementary Tables S10–12). Besides, for hybrid F1 carp, the HIF-1 signaling pathway, which was closely associated with adaptation to hypoxia, was significantly enriched.

After annotating DEGs, 162 genes were identified in two common carp strains and their hybrid F1 carp. Based on the enriched mutual GO terms and KEGG pathways, a total of eight DEGs were identified as core hypoxia-stressed common carp muscle genes, including *hsp70*, *hspa5*, *hsp47*, *cd276*, *tlr21*, *ddit4*, *cdkn1a*, and *gadd45ba*, and all of them were related to immune response or cellular stress response.

### DEGs of Two Common Carp Strains and Their Hybrid F1 Carp Liver Under Hypoxia

**FIGURE 3:**
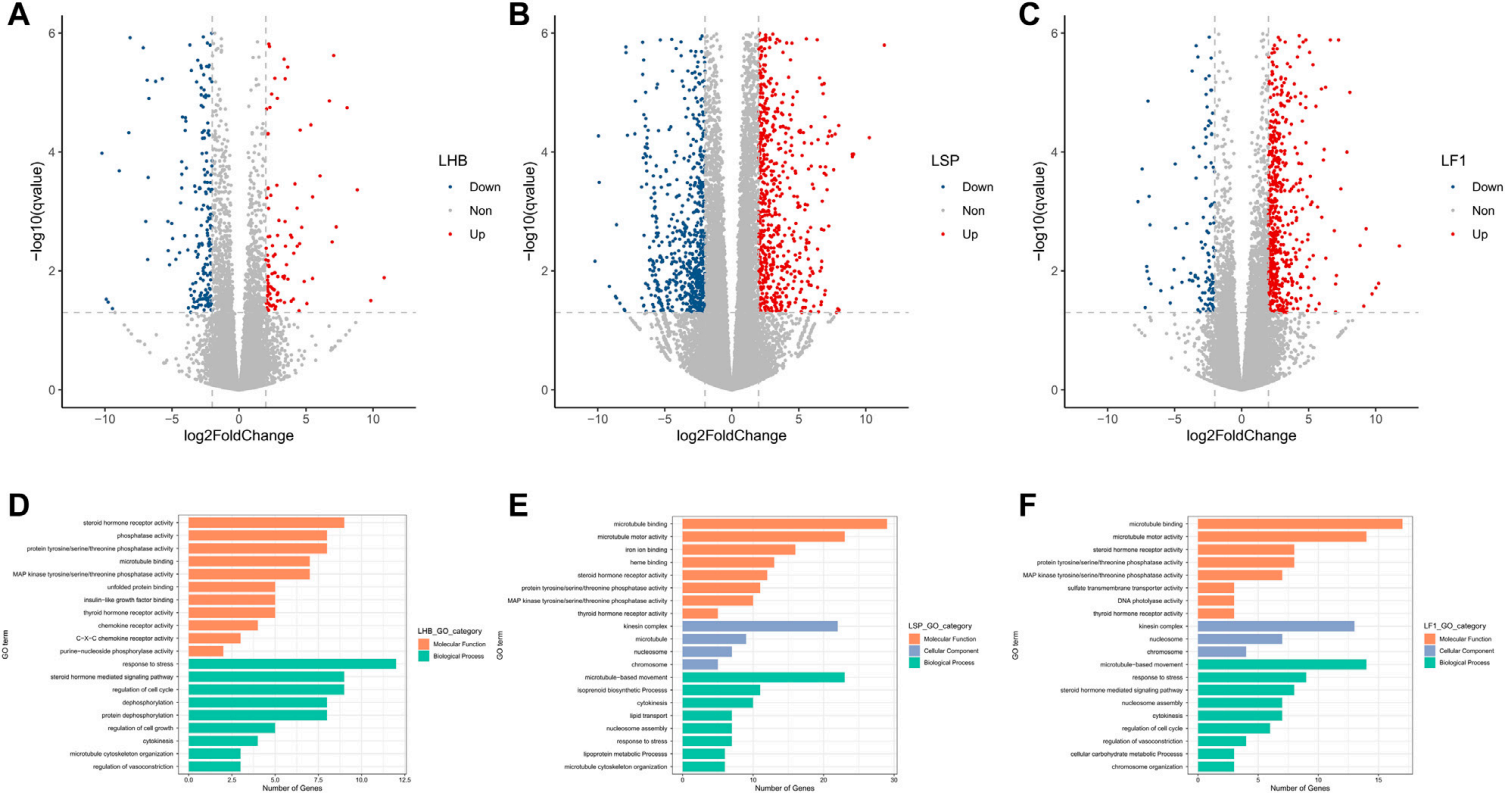
Volcano plot of log2 fold change and -log10 (FDR) of the differentially regulated genes demonstrated that most of the genes were up-regulated in all three strains of carp liver. **(A)** Volcano plot for Hebao red carp liver DEGs (LHBN1, LHBN2, LHBN3, LHBN5, LHBN6, LHBH1, LHBH2, LHBH3, LHBH4, LHBH5). **(B)** Volcano plot for Songpu mirror carp liver DEGs (LSPN1, LSPN3, LSPN4, LSPN5, LSPN6, LSPH1, LSPH2, LSPH3, LSPH5, LSPH6). **(C)** Volcano plot for hybrid F1 carp liver DEGs (LF1N1, LF1N2, LF1N3, LF1N4, LF1N5, LF1H1, LF1H3, LF1H4, LF1H5, LF1H6). GO enrichment analysis for genes differentially expressed between normoxia and hypoxia groups. **(D)**. Significantly enriched GO terms are shown for hypoxia-stressed Hebao red carp liver. **(E)**. Significantly enriched GO terms are shown for hypoxia-stressed Songpu mirror carp liver. **(F)**. Significantly enriched GO terms are shown for hypoxia-stressed hybrid F1 carp liver.

A total of 468 DEGs, including 349 up- and 119 down-regulated ones, were identified in the comparative between normoxic and hypoxic Hebao red carp liver, 1914 DEGs including 1157 up- and 757 down-regulated ones in Songpu mirror carp liver, and 812 DEGs with 686 up- and 126 down-regulated genes in hybrid F1 carp liver, respectively (Figures 3A,B,C; Supplementary Tables S13–15).

A total of 34 GO terms were significantly enriched in hypoxia-stressed Hebao red carp liver, and the most significant GO term was “response to stress” in the BP category (Figure 3D; Supplementary Table S16); “regulation of vasoconstriction,” which was related to hypoxia tolerance in the BP category was also enriched. Fifty-seven GO terms were significantly enriched in Songpu mirror carp liver, and two of them were related to oxygen binding and transport, including “iron ion binding” and “heme binding,” both in the MF category (Figure 3E; Supplementary Table S17). For hybrid F1 carp, the most significantly enriched GO term was “cytokinesis” in the BP category; and “regulation of vasoconstriction” in the BP category was also enriched (Figure 3F; Supplementary Table S18). “Response to stress” and “cytokinesis” both in the BP category, and “MAP kinase tyrosine/serine/threonine phosphatase activity,” “thyroid hormone receptor activity,” “steroid hormone receptor activity,” “protein tyrosine/serine/threonine phosphatase activity,” and “microtubule binding” in the MF category were all enriched in two common carp strains and their hybrid F1 carp liver, indicating that liver participated in hypoxia stress response by cellular stress response.

A total of 30, 29, and 28 KEGG pathways were significantly enriched in Hebao red carp, Songpu mirror carp, and hybrid F1 carp, respectively (Supplementary Tables S19–20). Human T-cell leukemia virus 1 infection, antigen processing and presentation, microRNAs in cancer, and osteoclast differentiation were enriched in two common carp strains and their hybrid F1 carp, suggesting that immune response plays essential roles in the common carp liver responding to hypoxia stress.

In addition, 135 mutual annotated DEGs in two common carp strains and their hybrid F1 carp were identified. Previous studies included *hsp70*, *hspa4a*, *hsp90*, *hsp47*, *gadd45ga*, *gadd45aa*, *cd276*, *tlr21*, *crtc2,* and *prc1a,* which were identified as core hypoxia-stressed common carp liver genes. In the end, 10 core DEGs are shown in Table 1.

**TABLE 1:** The core hypoxia-stressed genes in common carp.

*Gene_ID*	*Gene_name*	*Gene_annotation*
*HHLG5g0391*	*hsp70*	*Heat shock cognate 70-kd protein*
*HHLG9g1112*	*hspa5*	*Heat shock protein 5*
*HHLG29g0280*	*hsp47*	*Serpin peptidase inhibitor, clade H (heat shock protein 47), member 1b*
*HHLG26g0637*	*cd276*	*CD276 antigen-like*
*HHLG31g0383*	*tlr21*	*Toll-like receptor 21*
*HHLG27g0473*	*ddit4*	*DNA-damage-inducible transcript 4*
*HHLG43g0039*	*cdkn1a*	*Cyclin-dependent kinase inhibitor 1A*
*HHLG36g0310*	*crtc2*	*CREB-regulated transcription coactivator 2*
*HHLG50g0530*	*prc1a*	*Protein regulator of cytokinesis 1a*
*HHLG21g0552*	*gadd45ba*	*Growth arrest and DNA-damage-inducible, beta a*

### Hypoxia Stress Induces Different Gene Expression Patterns Among Two Common Carp Strains and Their Hybrid F1 Carp Muscle

#### Three Different Gene Expression Patterns Between Hebao Red Carp and Songpu Mirror Carp Muscle

We identified 2,049 DEGs including 918 up- and 1,131 down-regulated genes between Hebao red carp and Songpu mirror carp (HS, Hebao red carp was the control group) muscles under hypoxia stress and 1,651 DEGs including 813 up- and 838 down-regulated genes in the HS normoxia group, respectively (Supplementary Tables S22, 23). After annotating, all genes were divided into three types.

**FIGURE 4:**
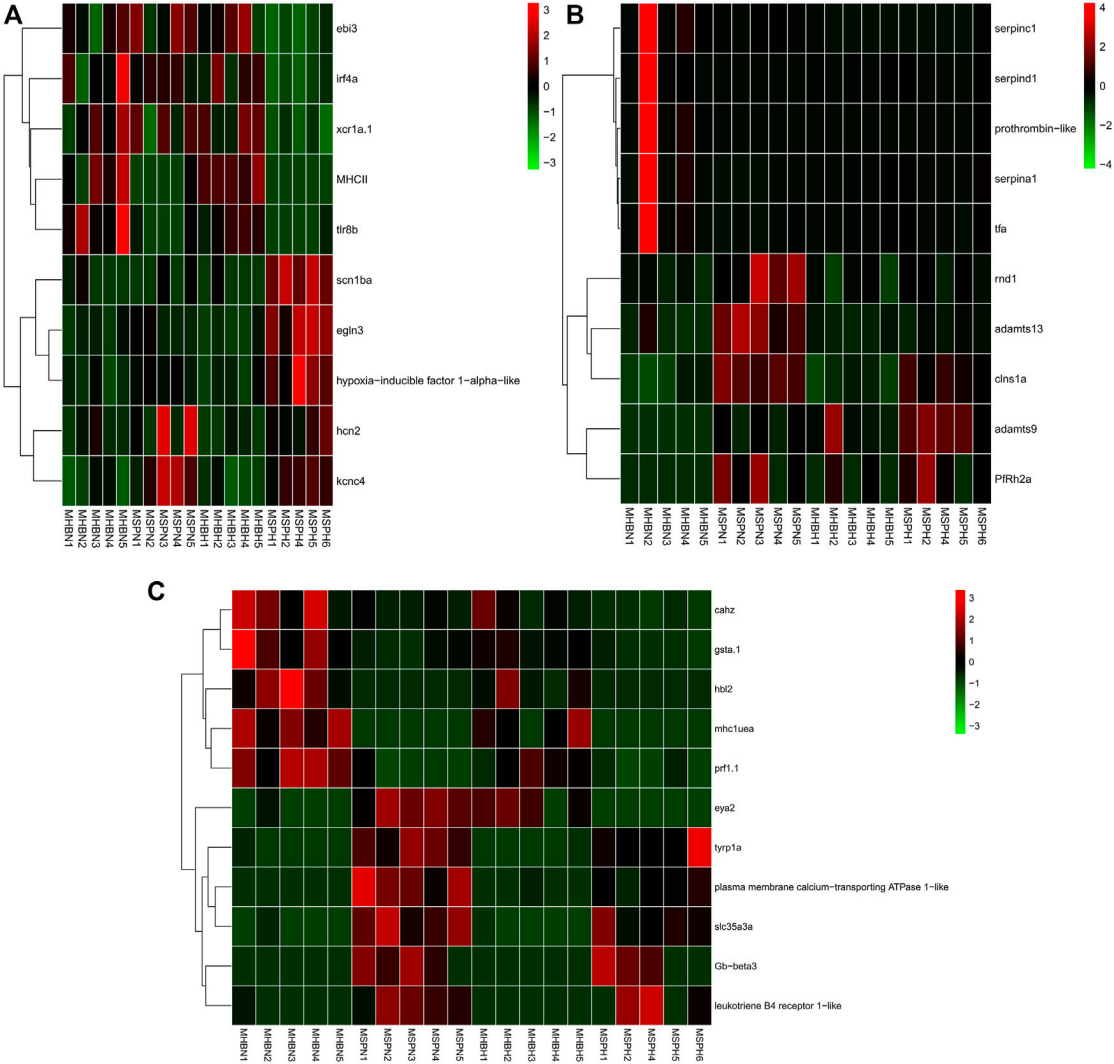
Hierarchical clustering of the three types of genes among three strains of carp muscle under hypoxia based on the average correlation of the log2 expression values. **(A)** HSDEGs. **(B)** NSDEGs. **(C)** HNDEGs (MHBN1, MHBN2, MHBN3, MHBN4, MHBN5, MHBH1, MHBH2, MHBH3, MHBH4, MHBH5; MSPN1, MSPN2, MSPN3, MSPN4, MSPN5, MSPH1, MSPH2, MSPH4, MSPH5, MSPH6).

The first type genes (hypoxia-specific differently expressed genes (HSDEGs)) were not significantly expressed in the normoxia group but were significantly up- or down-regulated under hypoxia stress, indicating that Hebao red carp and Songpu mirror carp had different regulation modes when responding to hypoxia. A total of 665 genes, including 284 up- and 381 down-regulated HSDEGs, were identified (Supplementary Table S24; Figure 4A). The up-regulated genes such as *egln3* (egl-9 family HIF 3) and HIF-1-alpha-like were identified. A total of 15 GO terms were enriched for up-regulated HSDEGs, among which three were significantly related to hypoxia tolerance, including “iron ion binding” and “heme binding” in the MF category and “voltage-gated sodium channel complex” in the CC category (Supplementary Table S25). A lot of down-regulated HSDEGs were related to immune response, such as *tlr8b* (Toll-like receptor 8b), *xcr1a* (chemokine (C motif) receptor 1a), *ebi3* (Epstein–Barr virus-induced 3), and *irf4a* (interferon regulatory factor 4a). Twenty-three GO terms were significantly enriched, and the most significant GO term was “immune response” in the BP category. Besides, there were four immune-related GO terms (Supplementary Table S26). The up-regulated HIF genes in Songpu mirror carp indicated that it had stronger hypoxia tolerance than those in Hebao red carp. On the contrary, the down-regulated immune-related genes in Songpu mirror carp showed that Hebao red carp mainly relied on its immune system responding to hypoxia stress.

The second type of genes (normoxic-specific differently expressed genes (NSDEGs)) were significantly up- or down-regulated in the normoxia group but were not significantly expressed under hypoxia stress. The NSDEGs would be regulated the same in two hypoxia-stressed carp strains, and 405 genes, including 214 up- and 191 down-regulated genes in this type, were identified (Supplementary Table S27; Figure 4B). The up-regulated genes such as *adamts13* (ADAM metallopeptidase with thrombospondin type 1 motif), *adamts9* (ADAM metallopeptidase with thrombospondin type 1 motif), and *PfRh2a* (reticulocyte binding protein 2 homolog A) were identified. Seventeen GO terms were significantly enriched for up-regulated NSDEGs, and the most significant term was “response to stress” in the BP category (Supplementary Table S28). The down-regulated NSDEGs, including prothrombin-like and *serpinc1* (serpin peptidase inhibitor, clade C (antithrombin), member 1), were identified. Among 13 significant GO terms for down-regulated NSDEGs, 4 of them were significantly related to oxygen transport, including “ferric iron binding” in the MF category and “iron ion transport,” “cellular iron ion homeostasis,” and “cellular iron ion homeostasis” all in the BP category (Supplementary Table S29). The down-regulated iron ion-related genes in Songpu mirror carp under normoxic indicated that Hebao red carp had stronger iron ion transport ability, which may play an important part in hypoxia tolerance.

The third type of genes (hypoxia-normoxic differently expressed genes (HNDEGs)) were significantly expressed in both normoxia group and hypoxia group, which were necessary for common carp normal physiological activities, so they were significantly expressed under any conditions. Finally, 257 genes were identified (Supplementary Table S30; Figure 4C). Three expression patterns were identified in HNDEGs, including up-regulated genes in two groups, down-regulated in two groups, and down-regulated in the normoxia group but up-regulated in the hypoxia group. The first expression pattern genes such as leukotriene B4 receptor 1-like and *Gb-beta3* (hemoglobin subunit beta) were identified. A total of 26 GO terms were significantly enriched for the first expression pattern genes, and 3 of them were involved in hypoxia tolerance, including “oxygen transport” in the BP category” and “oxygen binding” and “leukotriene receptor activity” both in the MF category (Supplementary Table S31). Twenty KEGG pathways were significantly enriched, among which 14 were involved in immune response, such as herpes simplex virus 1 infection and phagosome. The second gene expression pattern identified genes such as *hbl2* (hexose-binding lectin 2) and *mhc1uea* (major histocompatibility complex class I UEA). A total of 24 GO terms were enriched, and 7 of them were related to immune responses, such as “antigen processing and presentation” and “immune response,” both in the BP category (Supplementary Table S32). Three of the five enriched KEGG pathways were significantly associated with immune response, including inflammatory mediator regulation of TRP channels, MAPK signaling pathway, and NOD-like receptor signaling pathway. Moreover, the third expression pattern HNDEGs only included *eya2* (EYA transcriptional coactivator and phosphatase 2). A large part of the first expression pattern genes was involved in hypoxia tolerance, which indicated that Songpu mirror carp did have a stronger hypoxia tolerance ability than Hebao red carp under any conditions. The HNDEGs included a lot of immune-related genes suggesting the importance of immune response in physiological activities.

### Hypoxia Stress Induces Different Gene Expression Patterns Among Two Common Carp Strains and Their Hybrid F1 Carp Liver

#### Three Different Gene Expression Patterns Between Hebao Red Carp and Songpu Mirror Carp Liver

A total of 3283 DEGs including 1713 up- and 1570 down-regulated genes between Hebao red carp and Songpu mirror carp (HS, Hebao red carp was the control group) liver under hypoxia stress and 2687 DEGs including 1478 up- and 1209 down-regulated genes in the HS group under normoxic were identified by differential expression analysis, respectively (Supplementary Tables S36, 37). We used the same way to divide these genes.

**FIGURE 5:**
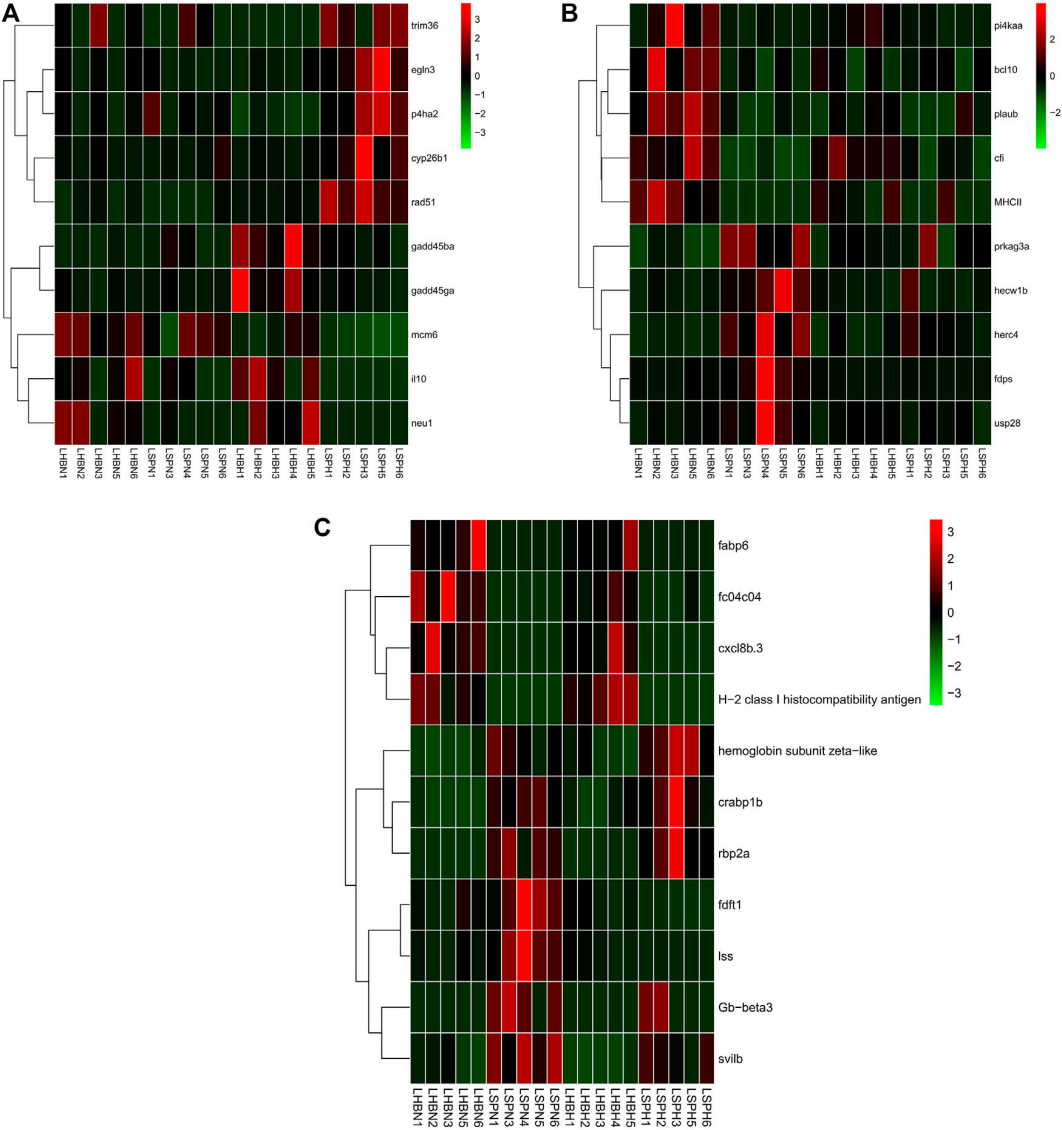
Hierarchical clustering of the three types of genes among three strains of carp liver under hypoxia based on the average correlation of the log2 expression values. **(A)** HSDEGs. **(B)** NSDEGs. **(C)** HNDEGs (LHBN1, LHBN2, LHBN3, LHBN5, LHBN6, LHBH1, LHBH2, LHBH3, LHBH4, LHBH5; LSPN1, LSPN3, LSPN4, LSPN5, LSPN6, LSPH1, LSPH2, LSPH3, LSPH5, LSPH6).

A total of 889 HSDEGs, including 440 up- and 449 down-regulated genes, were identified between Hebao red carp and Songpu mirror carp liver (Supplementary Table S38; Figure 5A). Up-regulated HSDEGs such as *egln3* (egl-9 family HIF 3) and *rad51* (RAD51 recombinase) were identified. Nineteen GO terms were enriched for up-regulated HSDEGs, among which the most significant GO term was “iron ion binding” in the MF category; another three GO terms were directly related to hypoxia tolerance, including “vasoconstriction” in the BP category and “heme binding” and “MAP kinase tyrosine/serine/threonine phosphatase activity” in the MF category (Supplementary Table S39). Among the 11 enriched KEGG pathways, hematopoietic cell lineage was enriched. A total of 26 GO terms were enriched for down-regulated HSDEGs such as *gadd45ga* (growth arrest and DNA-damage-inducible, gamma a) and *il10* (interleukin 10), among which five GO terms were related to cellular stress response, including “cytokine activity” and “DNA helicase activity” in the MF category (Supplementary Table S40). A total of 460 NSDEG genes including 283 up- and 177 down-regulated genes were identified, and 11 GO terms were enriched for up-regulated NSDEGs such as *prkag3a* (protein kinase, AMP-activated, gamma 3a non-catalytic subunit), and *usp28* (ubiquitin specific peptidase 28), among which “response to biotic stimulus” in the BP category was enriched (Supplementary Tables S41, 42; Figure 5B). Among 11 KEGG pathways, 6 were about immune response, such as NF-kappa B signaling pathway and RIG-I-like receptor signaling pathway. A total of 16 GO terms were enriched for down-regulated NSDEGs, including *cfi* (complement factor I) and *bcl10* (B-cell CLL/lymphoma 10), and the most significant GO term was “MHC class II protein complex” related to immune response (Supplementary Table S43). “Antigen processing and presentation” and “innate immune response” both in the BP category were also enriched. Two KEGG pathways were about the cellular stress response, including “regulation of apoptotic process” and “regulation of cell growth” both in the BP category. A total of 468 DEGs were identified for HNDEGs, among which 229 DEGs were up-regulated in both normoxic and hypoxic environments, 237 DEGs were down-regulated in both environments, and 2 DEGs (*fdft1* (farnesyl-diphosphate farnesyltransferase 1) and *lss* (lanosterol synthase (2,3-oxidosqualene-lanosterol cyclase)) were down-regulated under hypoxia stress but up-regulated in the normoxia group, respectively (Supplementary Table S44; Figure 5C). Among eight enriched GO terms for 239 up-regulated genes including *crabp1b* (cellular retinoic acid binding protein 1b), *Gb-beta3* (hemoglobin subunit beta), and hemoglobin subunit zeta-like, two were directly related to oxygen transport, including “oxygen transport” in the BP category and “oxygen binding” in the MF category (Supplementary Table S45). Among 12 GO terms enriched for 237 down-regulated genes such as *slc15a1b* (solute carrier family 15 (oligopeptide transporter), member 1b) and *cxcl8b.3* (chemokine (C–X–C motif) ligand 8b, duplicate 3), 2 were related to immune response, including “immune response” and “antigen processing and presentation”, both in the BP category (Supplementary Table S46).

A large part of the up-regulated HSDEGs was related to oxygen transport, indicating the stronger hypoxia tolerance ability of Songpu mirror carp. The NSDEGs, including many immune-related genes, proved that Hebao red carp mainly relied on the immune system in response to hypoxia stress. The up-regulated oxygen transport-related HNDEGs showed that Songpu mirror carp had a more active regulation mechanism of the respiratory.

### The HSDEGs’ Expression Patterns in Hypoxia-Stressed Hybrid F1 Carp

The HSDEGs were those Hebao red carp, and Songpu mirror carp had different regulation modes when responding to hypoxia. In order to understand the hybrid F1 carp’s heterosis, we mainly explored HSDEGs’ expression patterns in the hybrid F1 generation to explore whether the coping strategies of hybrid offspring under hypoxia stress tend to be paternal or maternal.

**FIGURE 6:**
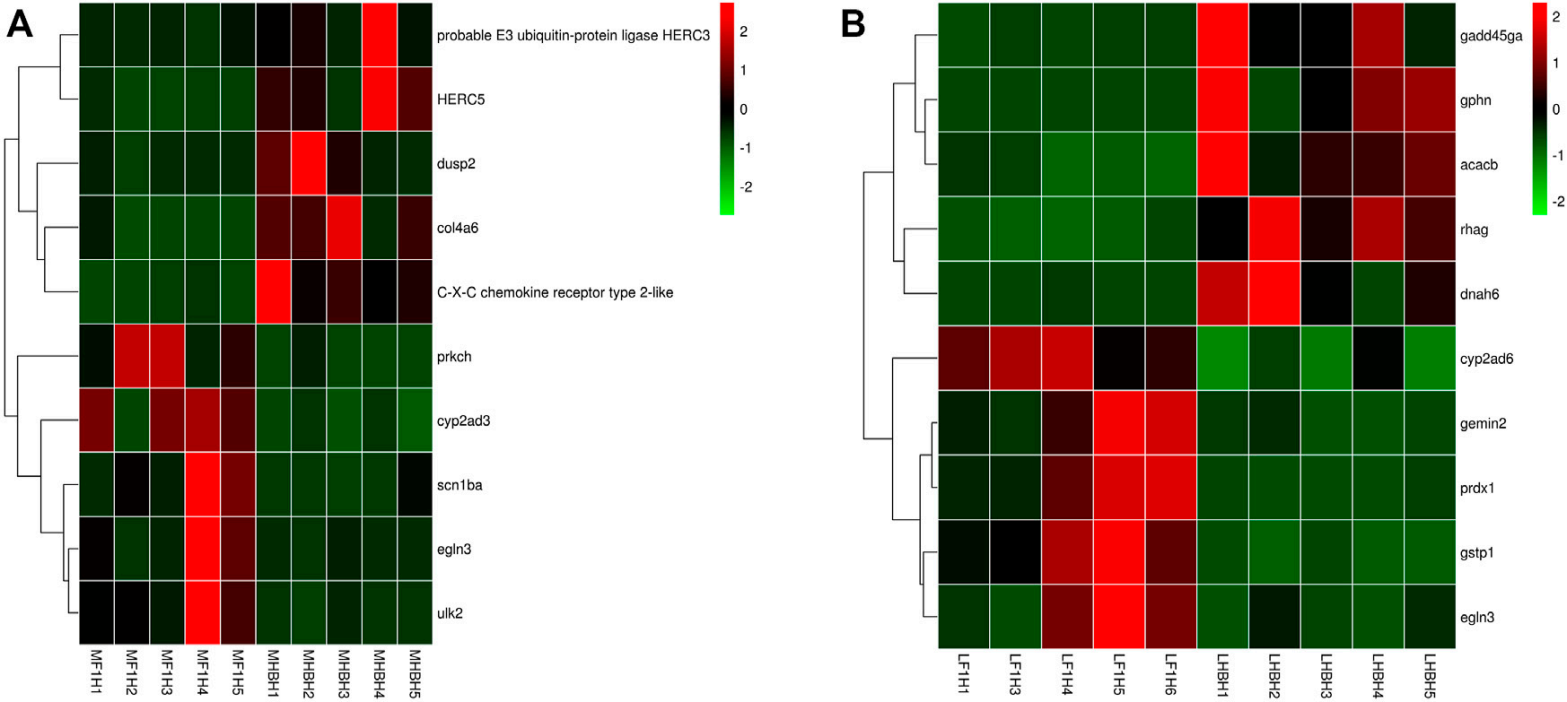
Hierarchical clustering of HSDEGs between hybrid F1carp and Hebao red carp based on the average correlation of the log2 expression values. **(A)** HSDEGs in the HF group muscle (MF1N1, MF1N2, MF1N3, MF1N4, MF1N5, MF1H1, MF1H2, MF1H3, MF1H4, MF1H5). **(B)** HSDEGs in the HF group liver (LF1N1, LF1N2, LF1N3, LF1N4, LF1N5, LF1H1, LF1H3, LF1H4, LF1H5, LF1H6).

A total of 87 HSDEGs were identified in the comparison between the hypoxic hybrid F1 carp and Hebao red carp muscle (Hebao red carp as the control group), and 40 of them were up-regulated in both two common carp strains (Songpu mirror carp and hybrid F1 carp) and 47 were both down-regulated (Supplementary Table S33). The up-regulated genes such as *egln3* (egl-9 family HIF 3) and *scn1ba* (sodium channel, voltage-gated, type I, beta a) were identified (Figure 6A). Two of the 13 significantly enriched GO terms were directly related to hypoxia tolerance, including “iron ion binding” and “heme binding,” both in the MF category, and the former was the most significant GO term (Supplementary Table S34). The down-regulated genes, including C–X–C chemokine receptor type 2-like, probable E3 ubiquitin-protein ligase HERC3 and H-2 class I histocompatibility antigen, and L-D alpha chain-like, were identified. Two of 17 enriched GO terms were related to immune response including “C–X–C chemokine receptor activity” in the MF category and “antigen processing and presentation” in the BP category (Supplementary Table S35). “MAP kinase tyrosine/serine/threonine phosphatase activity” in the MF category was also enriched. The up-regulated HIF genes in the hybrid F1 carp muscle showed that it had a more activated hypoxia response than Hebao red carp, and the latter was more dependent on immune response under hypoxia stress.

A total of 191 HSDEGs were identified in the HF group liver. Of the 191 HSDEGs, 129 HSDEGs were up-regulated and 62 HSDEGs were down-regulated (Supplementary Table S47). The up-regulated HSDEGs, including *egln3* (egl-9 family HIF 3) and *prdx1* (peroxiredoxin 1), were identified, and a total of 16 GO terms were enriched for them, among which the most significant term was “iron ion binding” in the MF category (Supplementary Table S48; Figure 6B). A total of 17 GO terms were enriched for the down-regulated HSDEGs, such as *gadd45ga* (growth arrest and DNA-damage-inducible), and the most significant GO term was “regulation of cell cycle” in the BP category. All these indicated that Hebao red carp mainly relied on cellular stress response and immune response to hypoxia stress (Supplementary Table S49).

### qPCR Validation Results

**FIGURE 7:**
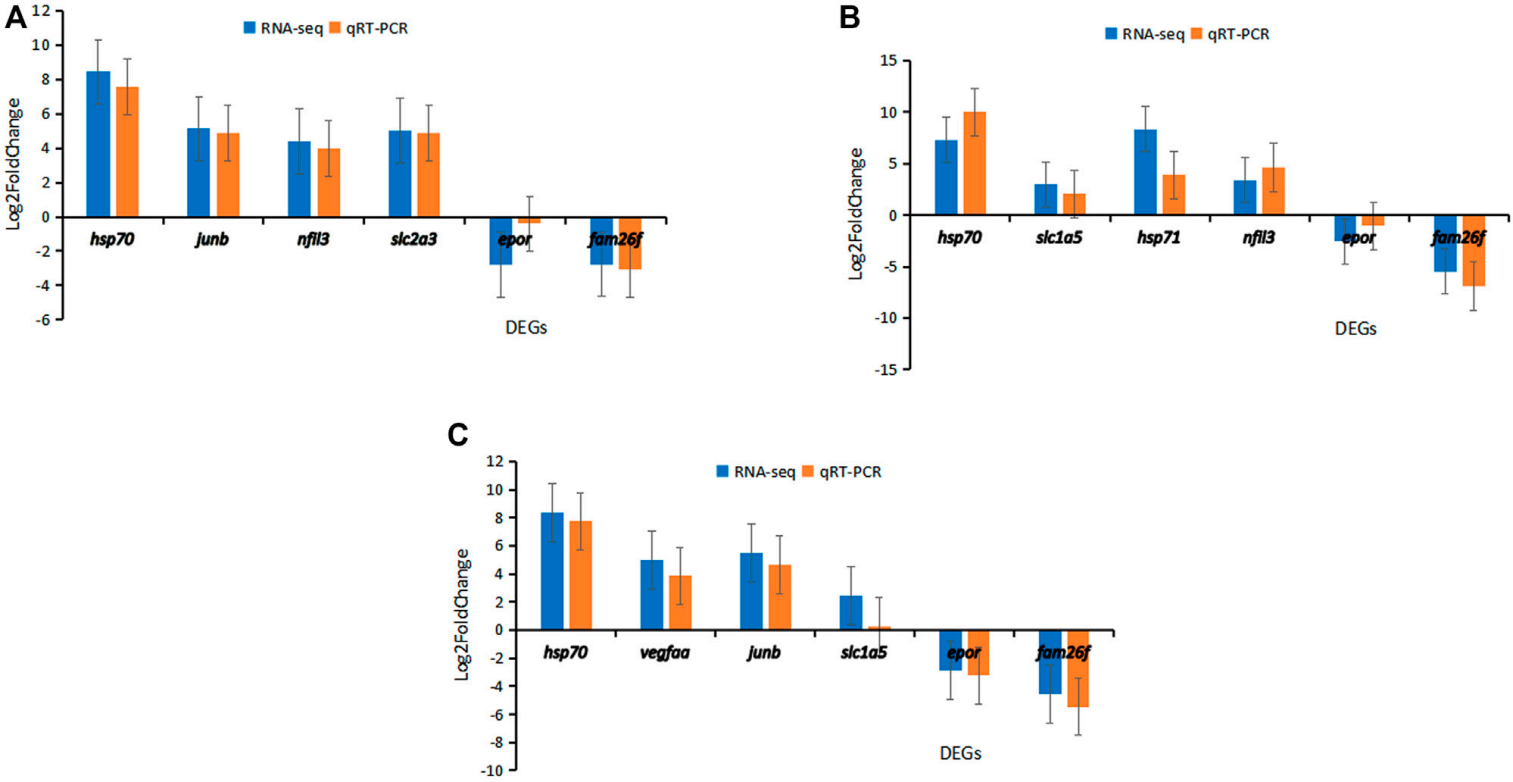
Results of qPCR validation for six DEGs in three strains of carp. **(A)** Hebao red carp muscle (MHBH1, MHBH2, MHBH3, MHBH4, MHBH5). **(B)** Songpu mirror carp liver (LSPH1, LSPH2, LSPH3, LSPH5, LSPH6). **(C)** Hybrid F1 carp muscle (MF1H1, MF1H2, MF1H3, MF1H4, MF1H5).

The qPCR results showed that *hsp70*, *junb*, *nfil3*, and *slc2a3* were up-regulated and *epor* and *fam26f* were down-regulated in Hebao red carp muscle (Figure 7A). In Songpu mirror carp liver, *hsp70*, *slc1a5*, *hsp71,* and *nfil3* were up-regulated and *epor* and *fam26f* were down-regulated (Figure 7B). In hybrid F1 carp muscle, *hsp70*, *vegfaa*, *junb*, and *slc2a3* were up-regulated and *epor* and *fam26f* were down-regulated (Figure 7C).

## Discussion

### Immune Response and Cellular Stress Response

Hypoxia is one of the most critical environmental factors influencing growth, immune response, reproduction, and fish survival (Xia et al., 2018). Besides, hypoxia in pond water can lead to disease outbreaks because it affects fish’s innate and adaptive immune response (Shoemaker et al., 2000; Boleza et al., 2001; Kvamme et al., 2013). *Tlr21* (Toll-like receptor 21) and *cd276* (CD276 antigen) were core hypoxia tolerance genes of two common carp strains and their hybrid F1 carp related to immune response. *Tlr21* was down-regulated in two common carp strains and their hybrid F1 carp muscle and two strains of carp liver but up-regulated in Hebao red carp liver, and it is a non-mammalian Toll-like receptor in fish species, which plays a critical role in innate immune when fish are faced with viral or bacterial pathogenic microbes (Li et al., 2018). In hypoxia-stressed Tibetan schizothoracine fish (*Gymnocypris eckloni*), *tlr21* was also down-regulated in the head kidney, spleen, and gill in response to hypoxia stress, which was consistent with our study (Qi et al., 2017). *Cd276* was also significantly regulated in two common carp strains and their hybrid F1 carp muscle and liver, and it can modulate T-cell-mediated immune response. When rabbitfish (*Siganus oramin*) was infected with the ciliate parasite *Cryptocaryon irritans*, *cd276* was significantly up-regulated to activate T cell (Jiang et al., 2019). The immune-related genes were expressed in two common carp strains and their hybrid F1 carp but expressed differently, and similar cases happened a lot. When large yellow croaker were under hypoxia stress, a large number of innate immunity genes were significantly down-regulated (Mu et al., 2020b); instead, in hypoxia-stressed catfish (*Ictalurus punctatus*) gill, two immune-related chemokine receptors (CXCR4b and CCR9a) were significantly up-regulated (Fu et al., 2017). Among the six mutual KEGG pathways in two common carp strains and their hybrid F1 carp muscle, four were related to immune response such as TNF signaling pathway, Kaposi sarcoma-associated herpesvirus infection, and two of the four mutual KEGG pathways in two common carp strains and their hybrid F1 carp liver were also associated with immunity, including human T-cell leukemia virus 1 infection and antigen processing and presentation. Based on our study and previous studies, the immune response does play an essential role in hypoxia-stressed fish, and it may be because the change of environment will induce the immune response firstly.

Cellular stress response happens when fish are under environmental pressures such as cold, heat, salinity, and hypoxia, which includes DNA damage and repair, cell cycle arrest, and (if the stress is out of threshold) cell apoptosis (Evans and Kültz, 2020). Moreover, we identified a lot of core hypoxia tolerance genes in two common carp strains and their hybrid F1 carp, which were related to cellular stress response, including *trim39* (E3 ubiquitin-protein ligase TRIM39-like), *gadd45g* (growth arrest and DNA damage-inducible protein GADD45 gamma), and *hsp70* (heat shock 70 kDa protein 1). The up-regulated *gadd45g* belongs to DNA damage-inducible gene family, and when cells are under stress, *gadd45g* will suppress cell growth and induce apoptosis (Ying et al., 2005). Hsp70 is a highly conserved stress protein that increases in response to stress for protecting cells (Morimoto et al., 1994; Hartl and Martin, 1995; Delaney and Klesius, 2004). In many mammals, *hsp70* is also stimulated in hypoxic conditions to resist hypoxic damage (Ferriero et al., 1990; Patel et al., 1995; Turman et al., 1997). Although protein hsp70 was not elevated in rainbow trout and chinook salmon (*Oncorhynchus tshawytscha*) cells when they were subjected to hypoxia, it was significantly increased in juvenile Nile tilapia (*Oreochromis niloticus*) blood cells, brains, and muscles exposed to hypoxia (Gamperl et al., 1998; Delaney and Klesius, 2004). Both ours and previous findings suggest that *hsp70* expression is species- and tissue-specific, contributing to differences in hypoxia tolerance in different species. Besides, two of four mutual GO terms in two common carp strains and their hybrid F1 carp muscle and one of seven mutual GO terms in liver were related to cellular stress response, including “regulation of cell cycle” and “response to stress,” and the latter was enriched in two tissues, indicating the importance of cellular stress response to hypoxia-stressed common carp.

### The Different Mechanisms of Hypoxia Tolerance in Two Common Carp Strains

Songpu mirror carp, as a selective strain of German mirror carp, is more tolerant of hypoxia (Li et al., 2008). The HSDEGs were in which Hebao red carp and Songpu mirror carp had different regulation modes when responding to hypoxia. Based on our study, “iron ion binding” and “heme binding” were significantly enriched for up-regulated HSDEGs. Iron and heme are closely related to oxygen transport; in some way, iron regulates heme. As a component of heme and iron–sulfur cluster-containing protein, iron is involved in oxygen transport and cellular respiration (Muckenthaler & Lill, 2012; Zhao et al., 2014). Our result proved that Songpu mirror carp had a more activated hypoxia stress response than Hebao red carp by its stronger ability to use iron ions, which ensured its physiological activities under hypoxia. “Heme binding” is also significant for oxygen transport in fish; the first and third steps of heme synthesis occur in mitochondria, and δ-aminolevulinate synthase (ALAS), which consists of ALAS1 and ALAS2, is needed here. While synthesizing ALAS2 is regulated by iron availability, when the iron is low, the synthesis of heme will be decreased; in a word, iron regulates the synthesis of heme, making influences on respiration (Wallander et al., 2006; Muckenthaler and Lill, 2012; Lathrop and Timko, 1993). The up-regulated iron ion-related pathways ensured the heme synthesis, which made greater influences on hypoxia-stressed Songpu mirror carp.

“MAP kinase tyrosine/serine/threonine phosphatase activity” was also enriched. MAP kinase is necessary to activate HIF-1 (HIF-1), which increases VEGF (vascular endothelial growth factor) expression, erythropoietin, GLUT-1 (glucose transport type 1), and glycolytic enzymes to protect hypoxia-stressed cells (Michiels et al., 2001; Kulisz et al., 2002; Emerling et al., 2005). In zebrafish, MAP kinase regulates the temporal expression of HIF-1α protein by regulating the H_2_O_2_ signal (Pelster & Egg, 2018). Our study identified HIF families such as up-regulated *egln3* (egl-9 family HIF 3)@ and protein HIF-1-alpha-like. HIFs are necessary for hypoxia-stressed fish, which can activate HIF-regulated genes when physiological and metabolic changes in organisms are prompted by hypoxic stress (Majmundar et al., 2010; Ortiz-Barahona et al., 2010). In acute and chronic hypoxia-stressed European sea bass (*Dicentrarchus labrax*), HIF-1α mRNA copies significantly increased (Terova et al., 2016). These hypoxia-related terms, pathways, and genes were significantly up-regulated in Songpu mirror carp, leading to the fact that Songpu mirror carp had more hypoxia tolerance. On the contrary, many down-regulated HSDEGs and pathways were related to immune and cellular stress responses, showing that Hebao red carp mainly relies on generalized physiological activities to adapt to hypoxia stress.

### The Heterosis of Hybrid F1 Carp Hypoxia Tolerance

As a tetraploid species, common carp has more forms of gene recombination and more degree of gene mutation than other fish. After hybridization, the genetic variation is further expanded; thus, the heterogeneity is expanded, so it is hopeful of obtaining heterosis (Xu et al., 2019). The genetic improvement of common carp is mainly focused on heterosis in the past. For growth, the Feng hybrid carp, a crossbreed of female Xingguo red carp and male mirror carp, has high heterosis for growth (Qiu, 1988). In order to improve the growth and survival of rohu carp (*Labeo rohita*), two complete diallel crosses with five stocks of common carp were processed (Gjerde et al., 2002). Moreover, there are also studies on heterosis obtained by hybridization to enhance the resistance of fish. For example, using a hybrid to improve the common carp resistance of koi herpesvirus (Ødegård et al., 2010), the hybrid of poeciliid fish *Xiphophorus maculatus* and *X. variatus* has more resistance of *Ichthyophthirius multifiliis* (Clayton and Price, 2010).

Based on our study, the expression patterns of all HSDEGs in hybrid F1 carp were consistent with those in Songpu mirror carp, indicating that the hypoxia-specific differently expressed genes’ regulation modes in hybrid F1 carp were more similar to Songpu mirror carp. The hypoxia tolerance-related genes and GO terms such as *egln3*, “iron binding,” and “heme binding” were identified in hybrid F1 carp. Our experiment also found that the hypoxia tolerance of hybrid F1 was stronger than that of Hebao red carp but lower than that of Songpu mirror carp, indicating the heterosis of hybrid F1 carp. Although the hybrid F1 of Hebao red carp and Songpu mirror carp needs more experimental verification in hypoxia tolerance, it still provides a new idea to further explore the molecular underpinnings of different common carp strains under hypoxia stress.

## Data Availability

The datasets presented in this study can be found in online repositories. The names of the repository/repositories and accession number(s) is PRJNA512071.
